# Physical activity as a predictor of fremanezumab response in chronic migraine – the Phy-Fre-Mig study

**DOI:** 10.1186/s10194-025-01965-w

**Published:** 2025-02-14

**Authors:** Álvaro Sierra-Mencía, Andrea Recio-García, David García-Azorín, Antonio José Molina de la Torre, Isabel Ros González, Ángel Luis Guerrero-Peral

**Affiliations:** 1 Headache Unit, Neurology Department, Hospital Clínico Universitario de Valladolid, Valladolid, Spain; 2 Neurology Department, Hospital Universitario Río Hortega, Valladolid, Spain; 3 Department of Medicine, dermatology and toxicology, Faculty of Medicine, University of Valladolid, Valladolid, Spain; 4 Área de medicina preventiva y salud pública, University of León, León, Spain

**Keywords:** Exercise, Sedentary behaviour, Migraine disorders, Antibodies, Monoclonal

## Abstract

**Background:**

The relationship between physical activity (PA) and migraine is insufficiently understood. Studies have not analysed levels of PA or Time Sitting (TS) during preventive treatment, nor the role these might play in the response to preventive treatment.

**Methods:**

An observational, longitudinal prospective study in a headache clinic was conducted. All consecutive chronic migraine patients initiating fremanezumab were invited to participate and were followed for three visits. The International Physical Activity Questionnaire (IPAQ) - long version was used.

**Results:**

Seventy-six patients with a median of 46 years old, 84.2% female were enrolled. One month after fremanezumab administration, there was a significant increase of most PA variables and a significant decrease in TS levels compared with baseline; headache days and walking, TS and migraine days showed a moderate correlation. Three months after initiation, all PA variables statistically increased and TS levels statistically decreased, and variables such as headache/migraine days showed a moderate correlation with all PA variables analysed. In the multivariate analysis, higher levels of walking at baseline were independently associated with response to fremanezumab (OR_a_: 1.194; CI: 1.018–1.401, *p* = 0.029).

**Conclusion:**

Patients who spent more time walking before starting treatment, were more likely to have a response to fremanezumab. PA and TS levels changed since the first month and correlated with clinical variables.

**Supplementary Information:**

The online version contains supplementary material available at 10.1186/s10194-025-01965-w.

## Introduction

Migraine affects 1 in 10 people worldwide [1]. It is also considered the world´s second cause of disability-adjusted life years (DALYs) of all neurological conditions among adults 20–59 [2]. Furthermore, it is the leading cause of disability in people under 50 years of age [3]. Migraine can cause an average loss of 3.2 days of work, 4.6 days of domestic activities and 2.1 days of social activities every three months [4]. Additionally, about 15% of people living with migraine report avoiding certain activities and commitments due to the fear of the next attack [5]. Moreover, migraine is typically aggravated by, or causes avoidance of routine physical activity, which indeed is listed as a diagnostic criterion [6]. Therapeutic management of migraine requires personalization of acute and preventive treatment approaches [7]. Fremanezumab, in pivotal clinical trials, revealed a favorable efficacy profile in the prevention of chronic migraine, being a safe, and well-tolerated monoclonal antibody [8]. Moreover, it has been shown to be effective, safe, and well-tolerated in the real-world setting, including in the chronic-resistant patients group [9].

Physical activity (PA) is defined as any corporal movement produced by skeletal muscles that results in energy expenditure [10]. The terms migraine and physical activity have seldom been studied together. Epidemiological research suggests that prevalence of migraine seems lower within patients who conduct regular PA [11, 12, 13]. However, these studies left several questions unanswered. Most of them evaluated PA solely during leisure activities, neglecting other domains, such as household, or commuting; and did not account for the intensity of PA. Additionally, the participants in these studies were often not diagnosed by headache specialists or account for migraine subtypes (e.g., chronic migraine). Another unresolved issue is, whether various clinical variables are associated with physical activity or time sitting (TS) levels. Finally, these studies have not assessed the role that the performance of PA or TS may have on treatment response.

Internationally validated questionnaires, such as the IPAQ, provide comprehensive tools for collecting reliable data across all domains and intensities of PA, as well as levels of TS. These instruments enable comparisons between studies and are particularly valuable for monitoring changes in PA and TS patterns within populations over time [14].

Thus, the aim of the study is to evaluate whether the physical activity and time sitting levels prior to the onset of fremanezumab are associated with a different probability of response. As secondary objectives, we aim to analyze if physical activity and time sitting levels change over the course of preventive treatment and if physical activity and time sitting levels are associated with clinical variables.

## Methods

### Study design

*The Fre-Phy-Mig* study is a single-centre, longitudinal prospective observational study. The study was performed in accordance with the principles of the declaration of Helsinki and was approved by the Valladolid East Clinical Research Ethics Committee PI 21-2408. All participants signed an informed consent form to participate in the study. The study was reported following the STrengthening the Reporting of OBservational studies in Epidemiology (STROBE) statement [15].

### Setting

The study was conducted in the headache outpatient clinic of the *Hospital Clinico Universitario de Valladolid*, Spain, a third-level, university, public hospital. Concerning the study period, data collection spanned from 30 July 2021 (first patient- first visit) to 03 July 2023 (last patient - last visit). All participants were followed for three months.

### Participants

The study population was composed of patients who started preventive treatment with fremanezumab (monthly), according to the criteria of the responsible physician and the local standard of care and clinical practice guidelines [16]. In the Spanish public healthcare system, CGRP monoclonal antibodies were subsidized in chronic migraine patients if they had previously failed to at least three migraine preventives, including onabotulinumtoxinA [16, 17]. Participants were included in the study if: (1) were aged between 18 and 65 years; (2) fulfilled criteria for chronic migraine (with or without aura) according to the International Classification of Headache Disorders (ICHD, 3rd version) [6]; (3) reported a migraine onset at least one year prior to the study onset; (4) were able to complete questionnaires. Patients were excluded if they: (1) had previously received another drug against CGRP; (2) presented other primary or secondary headache disorders, except for low-frequency tension-type headache or medication overuse headache; (3) had unusual migraine subtypes such as hemiplegic migraine (sporadic and familial), ophthalmoplegic migraine, migraine with neurological symptoms that are not typical of migraine aura (diplopia, altered consciousness or long duration); (4) had any clinically relevant cognitive or psychiatric disorder; (5) exhibited any pathology that could be a contraindication to physical exercise according to the guidelines of the Spanish Society of Sports Medicine (SEMED-FEMEDE) [18]; (6) were pregnant, expressed gestational desire, or breastfeeding; (7) were illiterate or had insufficient Spanish language performance.

Recruitment was done in the headache unit, where all consecutive patients were screened for eligibility and were invited to participate. Follow-up was conducted by in-person and telephone visits by healthcare providers.

### Variables

A structured clinical interview was performed by a trained-physician. Demographic and anthropometric variables included sex, age, and educational level, weight, height and body mass index (BMI). Prior and current history of psychiatric disorders and fibromyalgia was assessed. Clinical variables included the specific migraine diagnosis, according to the ICHD-3 (with/without aura; chronic migraine), age of onset, months of evolution of chronic migraine, the prior number of migraine preventive drugs classes, and the presence of medication overuse headache (MOH). Furthermore, headache days per month (HDM), migraine days per month (MDM), non-steroidal anti-inflammatory drugs (NSAIDs) and acetaminophen days per month (ADM), triptan days per month (TDM), and the median intensity of headache (MIH) episodes was measured in a 0–10 numeric verbal analogue scale (VAS) [19]. The adverse headache impact was assessed by the Headache Impact Test – 6 (HIT-6) [20]. Finally, physical activity and time sitting levels were evaluated using The International Physical Activity Questionnaire long-form (IPAQ-LF).

### Physical activity / exposure variable

The IPAQ-LF was adopted to collect data on Physical Activity (PA) and Time Sitting levels (TS) [21]. The tool quantifies physical activity in adults and can be used by either by telephone or self-administered [22]. The questionnaire has been previously translated and validated in the Spanish population [23]. The IPAQ collects data regarding the PA levels over the prior seven days. The information can be divided across five domains, including work, household, leisure-time, commuting, and time sitting. Each domain can be subdivided by the intensity of the activity thus we can distinguish between walking, moderate and vigorous. Information of each domain is collected using questions such as: “during the last 7 days, on how many days did you walk for at least 10 minutes at a time in your leisure-time?” and “How much time did you usually spend on one of those days walking in your leisure-time?”. IPAQ-LF requires between 15 and 30 min to be administered [22].

PA and TS levels were extracted and analysed following international guidelines [22]. Sum total of all domains (IPAQ-All domains) was obtained of the sum of all time invested by the participant in each domain (minutes/week). Leisure-time physical activity was obtained from the global computation of the sum of the three intensities of that domain (walking, moderate intensity, vigorous intensity) in minutes/week. Time spent walking was obtained from the global computation of the sum of the two domains: transportation and leisure-time (minutes/week). Time spent sitting (TS) was obtained from the average number of hours/day spent in a sitting or lying position. Total metabolic equivalent (METs) was calculated so that walking-intensity, moderate-intensity and vigorous-intensity accounted for 3.3, 4.0 and 8.0 METs, respectively. Thus, walking, moderate and vigorous MET-min/week were calculated by multiplying the selected MET value by the minutes/week of each intensity. The total PA MET-min/week was obtained by summing up the walking, moderate and vigorous MET-min/week score. A category analysis according to *Data processing guidelines of the IPAQ-LF* was considered, distinguishing category levels: low, moderate and high [24].

Finally, as workloads throughout the protocol changed and may be a confounding factor, work-domain physical activity and time sitting during week-days were dropped from the analysis. Moreover, calculations of bicycle use in transportation were not included, because no participants used this transport method through the study period. Only physical activities performed for at least 10 min per week were computed. The questionnaire was used in the hetero-administered form.

### Data sources /measurement

Data sources were clinical or telephone interview, using electronic and paper medical records. The survey data was collected on a standardized pre-defined template in paper format. The patients were diagnosed by headache neurologists. As per local standard of care and requirement for receiving CGRP mAb within the national healthcare system, all patients prospectively completed a headache diary, which included HDM, MDM, ADM, TDM, MIH. Intensity of headache episodes was evaluated according to VAS intensity rating scale, which consists in a 100-mm line with the end points of 0 (no pain) and 10 (worst pain) [19]. Response to fremanezumab was evaluated between weeks 8–12, compared with the month prior to the treatment use, as a reduction of ≥ 50% in the number of migraine days [25]. Furthermore, partial response was defined as a reduction of ≥ 30% in the number of migraine days between weeks 8–12 compared to baseline; and super-response was defined as a reduction of ≥ 75% in the number of migraine days between weeks 8–12 compared to baseline. The negative impact of headache was assessed by the Headache Impact Test – 6 (HIT-6), which includes six questions that are scored between 6 and 13, with a final score between 36 and 78. The score is stratified into four categories: little or no impact (< 50), some impact (50–55), substantial impact (56–59) and severe impact (> 59) [20].

### Intervention

The study period was divided in three prospective visits (further information is specified in the Supplementary Material): During baseline visit (T0), prior to any study procedures patients were informed about the study and were requested to provide consent to participate, clinical data was obtained from the headache calendars, and IPAQ-LF was administered. This T0 visit prior to the beginning of fremanezumab treatment.

The second and third visit took place one and three months after the fremanezumab administration (T1m and T3m, respectively). During these visits, clinical data was retrieved from headache calendars and the IPAQ-LF was administered.

### Bias

A series of biases were anticipated in the study design. Details are available in the Supplementary Material.

### Statistical methods and sample size

Descriptive statistics were used to examine the sociodemographic and clinical characteristics of the sample. Normal distribution of variables was tested by the Kolmogorov Smirnov test. Continuous quantitative variables were described as mean and standard deviation or median and interquartile range, depending on the type of distribution. Continuous variables in related groups were analysed using Wilcoxon test. For the correlations analysis between PA and clinical variables, the bilateral Spearman’s Rho test was used (Rs).

To assess the primary outcome and evaluate whether the levels of PA at baseline were associated with a different probability of response, a logistic regression analysis was done. The dependent variable was a 50% responder rate. First, a univariable regression analysis was conducted, and all the variables that showed a *P* value < 0.2 were included in a multivariable analysis. Odds ratios (OR) and their 95% confidence intervals (CI) are described. Due to the exploratory nature of the study, the substantial number of variables and the insufficient evidence to select the variables based solely on the literature, a stepwise multivariable regression analysis was done, employing the backwards procedure and the Wald method. We adjusted for multiple comparisons by using the False Discovery Rate (FDR) with Benjamini-Hochberg procedure [26]. In order to facilitate the data interpretation in the multivariate test, variables using minutes/week or minutes/day were converted to hours/week or hours/day. In all hypothesis tests performed, *p*-value < 0.05 was considered statistically significant.

Sample size calculation was based in another national study based on the same preventive-guidelines included 129 patients per year [17, 27]. Previous 21.3% increase in walking levels previously reported in a manuscript with equivalent population [28] was considered. Using a precision of 5.9% and a CI of 95% we obtained a final sample size of 76 patients. Concerning missing data, imputation methods by linear regression was used (clinical variables and physical activity variables at baseline and after three months had no missing data). The study analysis was performed as per protocol. For data analysis, the Statistical Package for Social Sciences (SPSS^®^, version 27.0, IBM Corp. Armonk, N.Y.) was used.

## Results

**Fig. 1:**
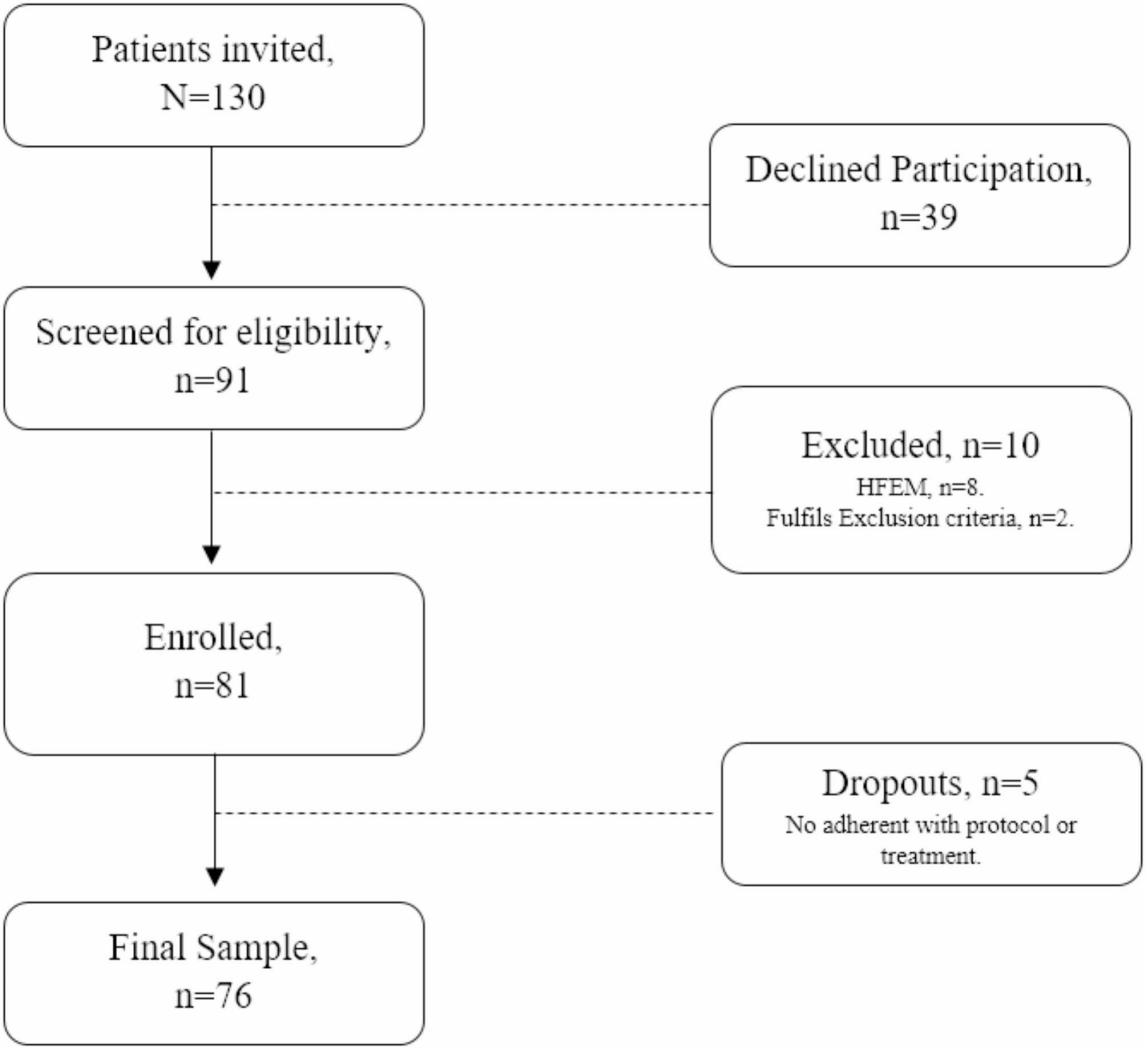
Flow diagram. Participant disposition

During the study period, 81 participants fulfilled the eligibility criteria and were enrolled and 76 completed the study. Patients flow diagram is detailed in the Fig. 1. Participants age was 46 [IQR: 38–55] years in median, and 64 (84.2%) participants were women. Concerning biometrics, median weight of patients was 68 [IQR: 57–81] Kg, median height 1.63 [IQR: 1.57–1.70] m, and BMI was 25 [IQR: 21–29] Kg/m^2^. Regarding education, 21 (27.6%) had a university degree.

Patients had prior history of psychiatric disorders in 32 (42.1%) cases and nine (11.8%) had prior history of physician-diagnosed fibromyalgia. Concerning migraine disease, the median age onset of migraine was 16 [IQR: 13–25] years old, with a median of 84 [IQR: 48–144] months of chronic migraine duration. Eighteen (23.7%) patients had migraine with aura. Patients had previously failed 5 [IQR: 4–6] preventive treatment classes. Regarding quality of life, patients reported a median baseline HIT-6 score of 67 [IQR: 64–72], reporting 68 (89.5%) of the sample severe impact.

### Treatment response

Table 1 shows the change in clinical variables during the study period. There was a statistically significant reduction in the median number of HDM, MDM, MIH, ADM, and TDM comparing baseline with the first month and third month after treatment administration (*p* < 0.001) (table 1). Partial response was achieved by 75% of the sample, 50% response by 63.2% of the sample and super-response by 28.9% of the sample.

**Table 1:** Clinical variables through the protocol

Variable	Baseline	T1m	T3m	FDR – adjusted *P* value
HDM (median, IQR)	23 [17–29]	16 [11–24]	11 [7–22]	*p* < 0.001
MDM (median, IQR)	15 [10–19]	9 [4–14]	5 [3–10]	*p* < 0.001
MIH (median, IQR)	7 [5.9–7.8]	6 [5-7.5]	6 [4.6–7.1]	*p* < 0.001
ADM (median, IQR)	15 [9–22]	11 [2–16]	7 [2–14]	*p* < 0.001
TDM (median, IQR)	9 [3–13]	4 [1–9]	2 [1–5]	*p* < 0.001
MOH (Percentage)	81.6%	43.4%	27.6%	*p* < 0.001

HDM: Headache days per month; MDM: migraine days per month (MDM); MIH: median intensity of headache; ADM: NSAIDs and acetaminophen days per month; TDM: triptan days per month

### Primary endpoint: Association between physical activity and fremanezumab response

In the univariable regression analysis, the PA variables that showed association with a higher probability of achieving a 50% response three months after fremanezumab onset were leisure-time physical activity, walking time and IPAQ-All domains. In contrast, time sitting and prior history of psychiatric disorders were associated with a lower odd of 50% response. Educational level showed a trend towards significance (*Table 2*).

In the multivariable analysis, the variables that remained associated with a higher probability of response to fremanezumab were time spent walking at baseline (OR_a_: 1.194; 95%CI: 1.018–1.401, *p* = 0.029; FDR – adjusted *p*-value = 0.029); and TDM (OR_a_: 1.120; 95%CI: 1.015–1.236, *p* = 0.024; FDR – adjusted *p*-value = 0.029). Prior psychiatric history (OR_a_: 0.242; 95%CI: 0.078–0.750, *p* = 0.014; FDR – adjusted *p*-value = 0.029) was associated with a lower probability of response.

**Table 2:** Univariable logistic regression analysis

Variable	OR_c_ - (95% CI)	*p*	FDR – adjusted *p*-value
Leisure-Time PA at baseline	1.216 - (1.019–1.450)	0.030	0.0525
Walking at baseline	1.189 - (1.029–1.374)	0.019	0.0525
IPAQ-All domains at baseline	1.071 - (1.008–1.137)	0.027	0.0525
TDM at baseline	1.084 - (1.002–1.172)	0.045	0.0525
Time Sitting at baseline	0.823 - (0.695–0.976)	0.025	0.0525
Psychiatric history	0.375 - (0.144–0.979)	0.045	0.0525
Educational level	0.723 - (0.500–1.044)	0.084	0.084

TDM: Triptan days per month, FDR: False Discovery Rate

### Changes in physical activity and time sitting levels during the study

**Fig. 2:**
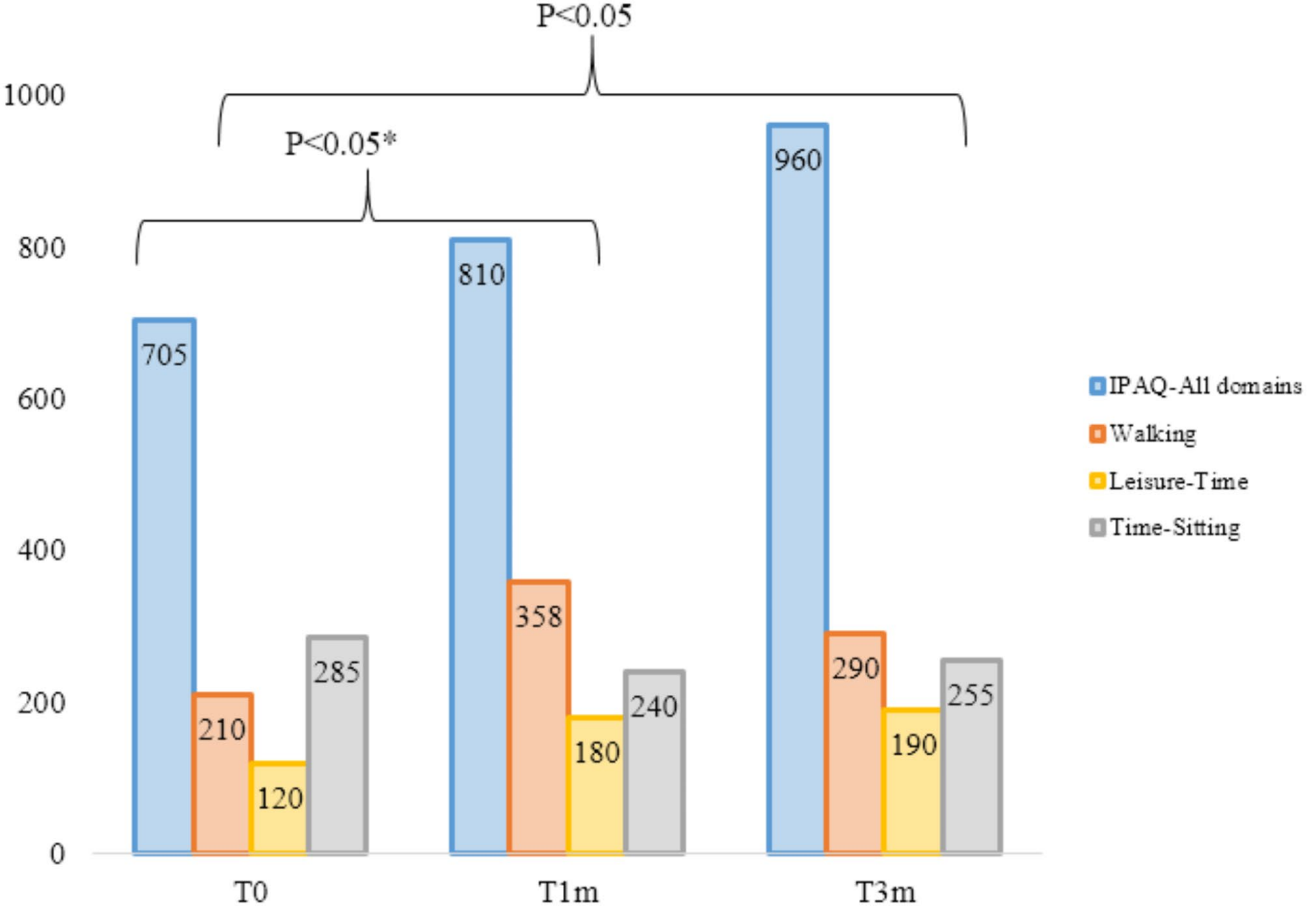
Physical activity and time sitting analysis through the protocol IPAQ-All-domains, walking, leisure- time are expressed in minutes/week TS levels are expressed in minutes/day *= All variables were statistically significant except for IPAQ-All domains between T0 and T1m (*p*=0.054)

Throughout the study period, physical activity and time sitting levels changed. One month after the fremanezumab administration (T1m) there was a statistically significant increase median of most PA variables and a significant median decrease in TS levels compared with baseline (*p* < 0.05). IPAQ-All domains did not show statistical significant increase compared to baseline. Three months (T3m) after fremanezumab onset, all PA variables showed a statistically significant increase compared to baseline (*p* < 0.05). TS median levels were significantly lower compared to baseline (*p* < 0.05). Results are summarized in Fig. 2. An analysis by intensities was performed throughout the study: At baseline (T0), low PA was performed by 9 (11.8%), moderate PA was performed by 18 (23.7%) and high intensity PA was performed by 49 (64.5%) of the sample. At T1m, low PA levels were performed by 11 (14.5%), moderate 12 (15.8%), high levels 53 (69.7%). Three months (T3m) after fremanezumab onset low PA was performed by 8 (10.5%), moderate PA was performed by 10 (13.2%) and high intensity PA was performed by 49 (76.3%). More detailed information is available in the Supplementary Material.

### Association between clinical variables and physical activity levels

There were no association between the physical activity, time sitting and clinical variables (frequency of headache/migraine or use of acute treatment) at baseline (*p* > 0.05).

**Fig. 3:**
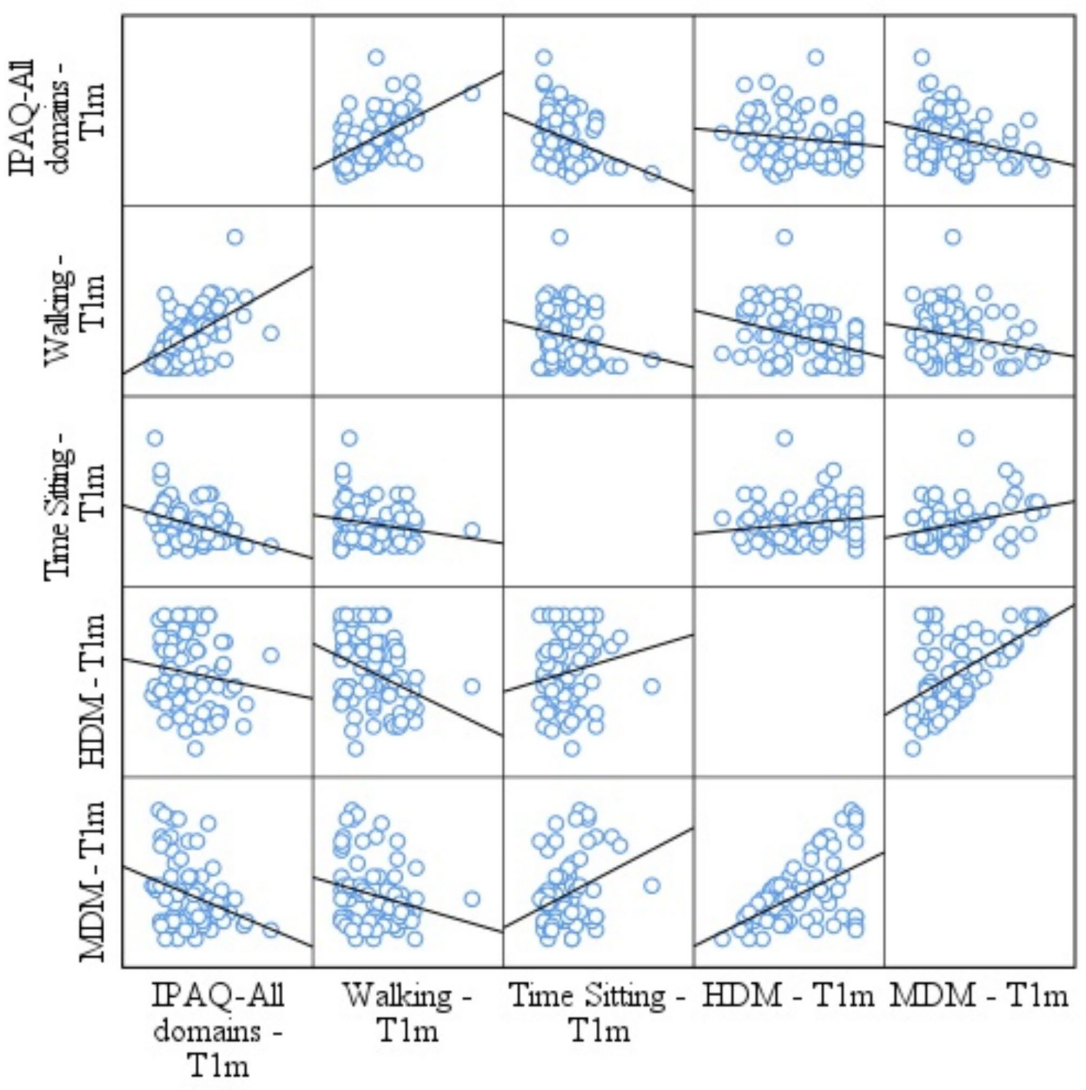
Scatter plot matrix analysis between clinical, PA and TS variables in T1m

One month after fremanezumab administration, a weak correlation between IPAQ-All domains and MDM (Rs -0,279; *p* = 0.015) was observed, a moderate correlation between walking and HDM (Rs -0.348; *p* = 0.002), and TS and MDM (Rs 0.319; *p* = 0.005). Figure 3 summarize the correlations in T1m.

**Fig. 4:**
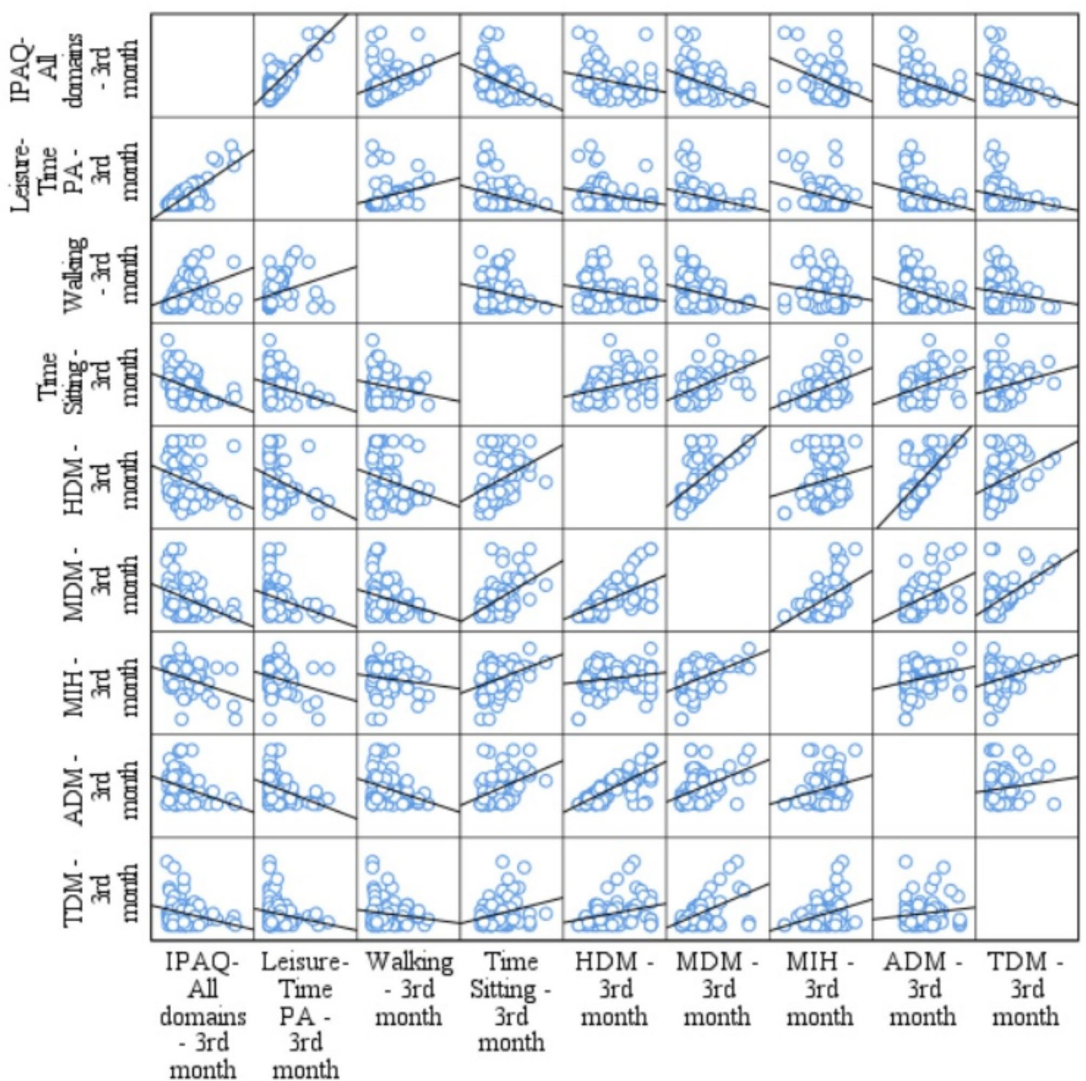
Scatter plot matrix analysis between clinical, PA and TS variables in T3m

Three months after fremanezumab onset, there was a statistically significant moderate correlation between IPAQ-All domains and HDM (Rs -0.355; *p* = 0.002), MDM (Rs -0.419; *p* < 0.001), MIH (Rs -0.316; *p* = 0.005), ADM (Rs -0.354; *p* = 0.002). There was a statistically significant moderate correlation between leisure-time PA and HDM (Rs -0.341; *p* = 0.003), MDM (Rs -0.334; *p* = 0.003), ADM (Rs -0.358; *p* = 0.002). Walking activity also showed a weak correlation HDM (Rs -0.222; *p* = 0.050), and ADM (Rs -0.236; *p* = 0.040). Time sitting showed a strong correlation with MDM (Rs 0.520; *p* = < 0.001), a moderate correlation with HDM (Rs 0.434; *p* < 0.001) and MIH (Rs 0.351; *p* = 0.002), ADM (Rs 0.424; *p* = < 0.001), and a weak correlation with TDM (Rs 0.247; *p* = 0.031). Results in T3m are summarized in Fig. 4.

## Discussion

The current study examined whether physical activity (PA) and time sitting (TS) levels could be somewhat predictive of response to fremanezumab in treatment-resistant chronic migraine patients. The main findings were that physical activity performing at baseline was associated with a higher probability of response to preventive treatment. Moreover, patients showed a statistically significant increase in their total physical activity, leisure time and walking time since the first month after treatment onset and time sitting levels decreased. Finally, physical activity parameters showed correlation with multiple clinical variables, supporting the interplay between patients’ activity and their clinical situation. These findings reinforce the importance of counseling patients about the physical activity practice as a healthy habit.

Doing PA regularly, as well as avoiding TS, are well established health-related recommendations and have been associated with better quality of life [29, 30], not only in the short term, but also in the medium and long term, even in patients with neurological diseases [31]. The World Health Organization 2020 guidelines on physical activity and sedentary behavior state that all adults should regularly undertake physical activity and that some physical activity is better than none [32]. This guideline emphasizes that physical activity confers benefits for many health markers, including mental health outcomes, such as reduced symptoms of anxiety and depression, cognitive health and sleep [32], conditions which are frequently comorbid in chronic migraine patients [33].

Despite this, available data shows that migraine patients perform less PA [34, 35]. One epidemiological study performed in Spain, included 17,139 individuals with a mean age of 46 years. This study, handled data from the national health survey in Spain (2017), through IPAQ-Short Form and categorize PA levels according to their validated criteria [34]. Similarities with our results can be found in the fact that, people who performed fewer PA had a higher prevalence of migraine than active and very active people (*p* < 0.05), which may be correlated to the clinical situation of the patient, rather than show a causal link [34]. Population categorization population into intensities (low, moderate and high) differs. A possible explanation for these facts, lies on the in the different cut-off points between the two studies. In addition, since 2017, Spain has participated in urban planning improvements, potentially making patients more friendly to PA practice [36]. Another study performed in Brazil (ELSA-Brasil - cohort) and evaluated the relationship between PA and migraine, used the IPAQ-LF questionnaire. The study divided PA time by domains or intensities, showing that higher levels of physical inactivity were associated with higher migraine frequency [35]. This study could not clearly identify correlation between the patient’s clinical situation and the performance of PA, maybe because patients included were not diagnosed by a neurologist with expertise in headache disorders. Furthermore, it is possible that our sample, being part of the same health area, had a more homogeneous environment and we have been able to demonstrate a certain relationship between the clinical situation and the performance of PA and a less TS [37].

To date, there is not much evidence that has studied PA in headache patients with a longitudinal design in headache units [28, 38, 39]. The most comparable study evaluated total walking time in chronic migraine patients using an alternative measurement method (patients’ smartphones) before and after fremanezumab use [28]. Equivalent findings were reported, demonstrating a significant increase in steps/day after three months of the beginning of the treatment. As well as steps/day before treatment initiation were correlated with an increased likelihood of response to fremanezumab [28]. Others studies with a case-control design, showed that migraine patients took fewer steps per day than healthy controls [38, 39]. To our knowledge, no other studies have specifically assessed TS levels in this patient population.

Performing physical exercise is consistently recommended by experts and supported by the existing literature in migraine field [40]. Most of published systematic-reviews and meta-analysis of intervention studies, link physical exercise with an improvement in clinical variables [41, 42, 43]. However, most interventional studies have not been conducted in chronic-resistant patients, leaving this population without clear evidence. Effective patient-healthcare provider communication is essential, recognizing individual factors influencing migraine onset and management [44]. Prescription of therapeutic exercise in migraine, appears to be a safe and effective intervention that could decrease migraine symptoms and disability and increase quality of life [45]. Physiotherapists and other exercise specialist, focused in chronic pain and therapeutic exercise prescription, play a crucial role in implementing exercise programs and educating patients about movement. These physiotherapists, also trained in motivational interviewing, are able to guide patients in practicing safe and effective exercises, adjusting the treatment plans according to the patient’s physical and emotional responses [46]. This recommendation should be made considering individualized and personalized patient’s preferences, by taking into account the multitude of possibilities for the PA realization, such as: aerobic exercise, leisure walking, yoga [43].

Migraine, contradictory to other common primary headache types, is characterized by the aggravation of symptoms by routine physical activity. Consequently, adults with migraine may avoid physical activity during attacks [28]. Migraine patients sometimes report physical exercise as a pain trigger [47] and therefore some of them may adopt PA avoidance strategies [48]. Prodromal symptoms, such as fatigue, nausea, photophobia, and phonophobia, may impair the ability to engage in physical activity too [47, 49]. Thus, studies have shown that clinical improvement also includes prodromal and associate symptoms, other than pain [50], so patients who benefit from preventive therapies could end up increasing their PA levels and reducing their time sitting. Furthermore, from a molecular perspective, significant elevations in serum CGRP levels have been observed during endurance exercise, and these increases were dependent on exercise intensity [51]. Therefore, if CGRP is inhibited through the preventive action of fremanezumab, it is plausible that PA would not provoke a headache which could lead to a migraine [52].

Multiple factors associated with treatment response to monoclonal antibodies have been examined by various studies [53, 54]. Variables such as depression, obesity and medication overuse headache, have shown to be negatively related with response to monoclonal antibody therapy [53]. Several of these factors have also demonstrated a strong inverse relationship with PA performance [55, 56]. One possible explanation for these findings could be the motivational aspect that connects physical activity engagement with the experience of migraine pain [57].

Although the sample was drawn from patients diagnosed by a headache-physician, attending outpatient clinics at a tertiary hospital, several limitations must be considered when interpreting the results. First, the sample size was modest. Second, self-selection bias could somewhat be mitigated by the screening of consecutive patients initiating treatment. Third, PA and TS were self-reported through the IPAQ-LF questionnaire and not collected from an accelerometer or pedometer device that track patients’ more objective PA. Due to observational design, this results should be evaluated with prudence: The presence of other factors, such as, motivation to perform PA secondary to a better clinical situation has not been considered. Moreover, the risk of type I error and overfitting of the results due to the use of a Backwards multivariable regression analysis. Our results should be replicated in future studies, experimental, randomized, control group design is recommended to adequately evaluate this relationship and other statistical methods might be selected now that our study has already provided some evidence to guide the selection of variables.

In conclusion, patients who performed more physical activity prior to receiving fremanezumab had a higher probability of responding to the treatment, while patients who spent more time sitting had fewer odd of response. Physical activity levels increased over the course of fremanezumab, and time sitting decreased since the first month. Physical activity and time sitting showed correlation with multiple clinical variables, supporting that patients’ physical activity may be linked to their clinical situation.

## Electronic supplementary material

Below is the link to the electronic supplementary material.

Supplementary Material 1

## Data Availability

No datasets were generated or analysed during the current study.
