# Supplementary material for: Physical activity as a predictor of fremanezumab response in chronic migraine – the Phy-Fre-Mig study
Source: J Headache Pain. 2025 Feb 14;26(1):34. doi: 10.1186/s10194-025-01965-w (PMC11827165; doi:10.1186/s10194-025-01965-w)
Supplement: Supplementary file 1 — Supplementary Material 1 [file 10194_2025_1965_MOESM1_ESM.docx]

**Supplementary Material**

*Supplementary Graphic 1 - Intervention / Study visits*


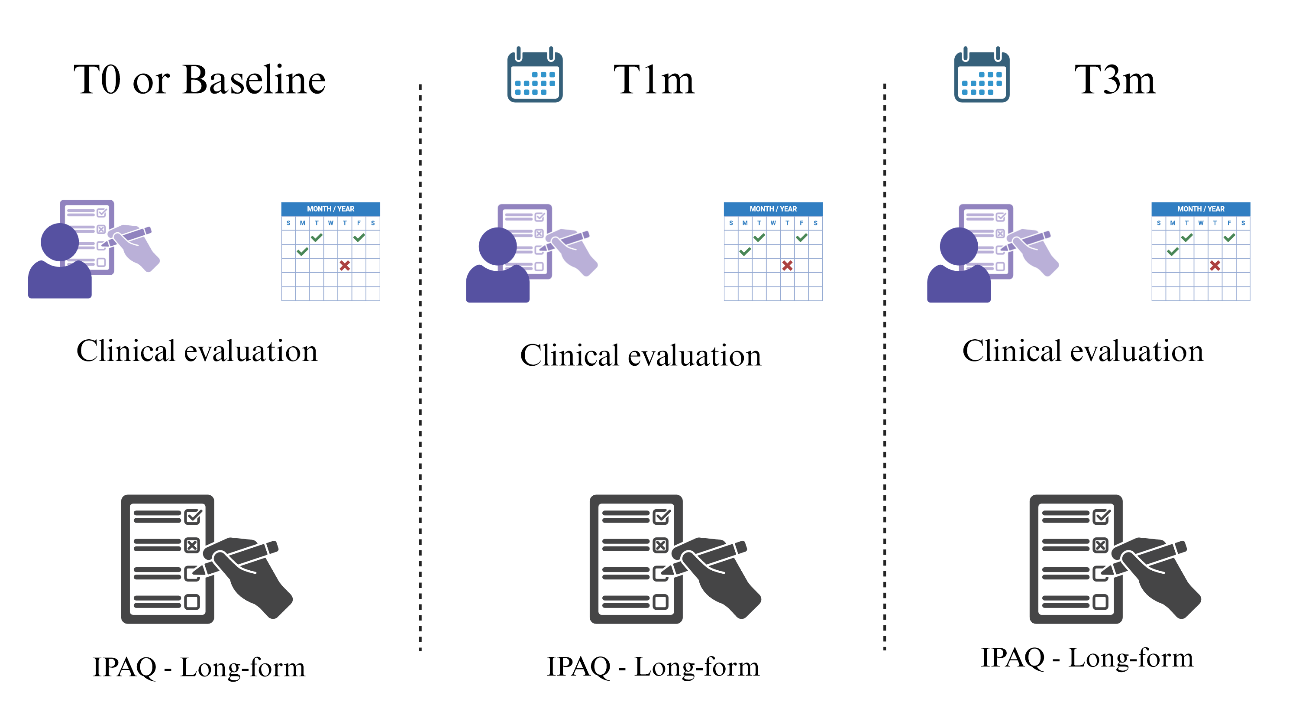


**Graphic 1.** Scheme of the study visits.

*Study bias*

Information bias was minimized through the standardized collection of all study variables. Ascertainment of exposure was assessed through a structured interview and the use of electronic health records. Recall bias was prevented using headache diaries in paper format. Social desirability bias was mitigated by repeating the assessment of physical activity levels 2 times through the protocol. Voluntary bias was mitigated including all consecutive patients that initiate preventive treatment with Fremanezumab according local guidelines.

*Supplementary Figure 1 - Physical activity and time sitting levels during the study – Intensity Analysis*


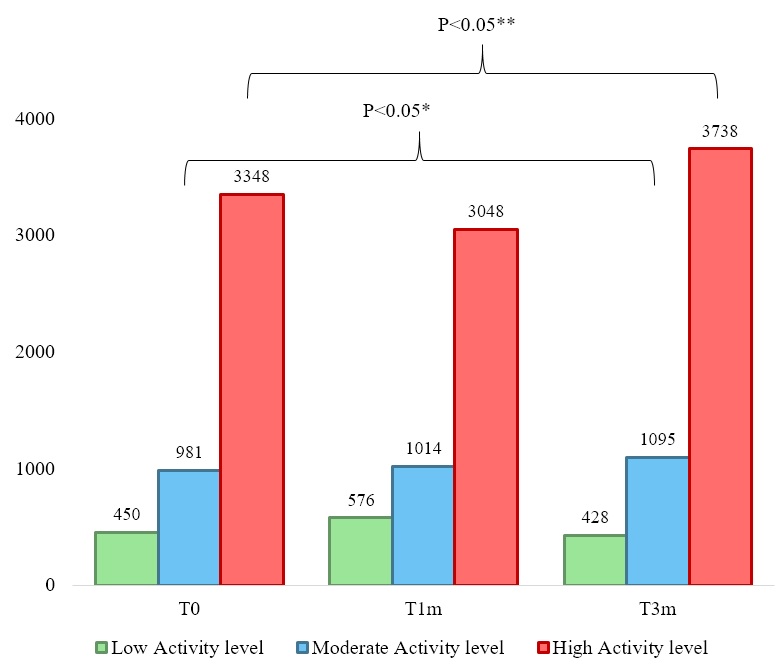


**Figure 1. Physical Activity and Time Sitting analysis by intensities.**

All values are expressed in METs/min/week.

*= Moderate intensity between T0-T3m was statistically significant (p=0.004).

All

**=High activity levels were statistically significant between T0-T1m (p=0.045)

and T0-T3m (p=0.008).

*Supplementary Table 1 - Multivariate analysis – All variables*

| **Variables at baseline** | **OR - (95% CI)** | **p** |
| --- | --- | --- |
| Aura | 0.658 - (0.224 – 1.929) | 0.446 |
| Age of migraine onset | 0.974 - (0.928 - 1.023) | 0.300 |
| Months of evolution of chronic migraine | 1.000 - (0.994 - 1.006) | 0.971 |
| HDM | 0.988 - (0.910 - 1.073) | 0.777 |
| MDM | 0.987 - (0.910 - 1.071) | 0.759 |
| MIH | 0.941 - (0.664 - 1.333) | 0.732 |
| ADM | 0.978 - (0.926 - 1.034) | 0.432 |
| **TDM** | **1.084 - (1.002** - **1.172)** | **0.045*** |
| MOH at baseline | 1.364 - (0.419 - 4.435) | 0.606 |
| HIT-6 at baseline | 0.985 - (0.908 - 1.068) | 0.709 |
| HIT-6 – Severe Impact (>59) | 0.538 - (0.101 - 2.870) | 0.468 |
| Previous preventives classes | 0.854 - (0.650 1.122) | 0.258 |
| **Psychiatric History** | **0.375 - (0.144 – 0.979)** | **0.045*** |
| Fibromyalgia | 0.418 - (0.102 1.710 | 0.225 |
| Age | 1.002 - (0.960 1.045 | 0.932 |
| Sex | 1.923 - (0.474 7.797 | 0.360 |
| **Educational level** | **0.723 - (0.500 - 1.044)** | **0.084*** |
| BMI | 0.987 - (0.896 1.086 | 0.782 |
| **Leisure-Time PA** | **1.216 - (1.019 - 1.450)** | **0.030*** |
| **Walking** | **1.189 - (1.029 - 1.374)** | **0.019*** |
| **IPAQ-All domains** | **1.071 - (1.008 - 1.137)** | **0.027*** |
| **Time Sitting** | **0.823 - (0.695 - 0.976)** | **0.025*** |

**Table 1.** Univariable logistic regression analysis. All variables analyzed.

HDM: Headache days per month; MDM: Migraine days per month; MIH: Median intensity of headache; NDM: Non-steroidal anti-inflammatory drugs days per month; TDM: Triptan days per month; MOH: Medication Overuse Headache.
